# The role of lipid species in membranes and cancer-related changes

**DOI:** 10.1007/s10555-020-09872-z

**Published:** 2020-04-20

**Authors:** Tore Skotland, Simona Kavaliauskiene, Kirsten Sandvig

**Affiliations:** 1 Department of Molecular Cell Biology, Institute for Cancer Research, Oslo University Hospital, Ullernchausséen 70, 0379 Oslo, Norway; 2 Department of Biosciences, University of Oslo, 0316 Oslo, Norway

**Keywords:** Caveolae, Membrane domains, Endocytosis, Leaflet interdigitation, Molecular dynamic simulation, Cancer

## Abstract

Several studies have demonstrated interactions between the two leaflets in membrane bilayers and the importance of specific lipid species for such interaction and membrane function. We here discuss these investigations with a focus on the sphingolipid and cholesterol-rich lipid membrane domains called lipid rafts, including the small flask-shaped invaginations called caveolae, and the importance of such membrane structures in cell biology and cancer. We discuss the possible interactions between the very long-chain sphingolipids in the outer leaflet of the plasma membrane and the phosphatidylserine species PS 18:0/18:1 in the inner leaflet and the importance of cholesterol for such interactions. We challenge the view that lipid rafts contain a large fraction of lipids with two saturated fatty acyl groups and argue that it is important in future studies of membrane models to use asymmetric membrane bilayers with lipid species commonly found in cellular membranes. We also discuss the need for more quantitative lipidomic studies in order to understand membrane function and structure in general, and the importance of lipid rafts in biological systems. Finally, we discuss cancer-related changes in lipid rafts and lipid composition, with a special focus on changes in glycosphingolipids and the possibility of using lipid therapy for cancer treatment.

## Introduction

The plasma membrane (PM) of eukaryotic cells consists of a bilayer of lipids and many membrane-embedded proteins. The membrane lipids are grouped into different classes as shown in Table 1. Most of this classification is based on the different head groups of these lipids. Addition of fatty acyl chains with different number of carbon atoms and double bonds gives rise to the many species in each lipid class, summing up to that cellular membranes probably contain several thousand lipid species. The PM has an asymmetric distribution of lipids in the two leaflets, with most of the phosphatidylcholine (PC) and probably all sphingolipids located in the outer leaflet, whereas the other PM lipids, such as phosphatidylserine (PS), phosphatidylethanolamine (PE), and phosphatidylinositol (PI), are located in the inner leaflet [1, 2]. The hydrophilic heads of these lipids are facing the hydrophilic surroundings on both sides of the membrane, whereas the hydrophobic chains are in the middle of the membrane. The membrane lipids and the membrane-embedded proteins form various subdomains, thus giving rise to a large heterogeneity of the PM. The areas often referred to as lipid rafts (now also referred to as membrane rafts) are enriched in cholesterol (CHOL), which constitutes 30–40% of the lipids in the PM, and sphingolipids [3–6]. Sphingomyelin (SM) is the dominating sphingolipid in the cell and in the PM, but there are also variable amounts of glycosphingolipids which are divided into many subclasses with different carbohydrates in their head groups, including the neutral globosides and the negatively charged gangliosides, as reviewed in [7]. We refer to a new and detailed review article describing the diversity of membrane lipid composition [8], and will here just briefly comment on this diversity.

**Table 1:** Most common lipid classes in biological membranes

Lipid class/abbreviation	R1	R2	Headgroup
Phosphatidylcholine/PC LysoPC/LPC^b^ Ether-linked PC^c^	FA^a^ FA Alkyl, alkenyl	FA H FA	Choline
Phosphatidylserine/PS	FA	FA	Serine
Phosphatidylethanolamine/PE	FA	FA	Ethanolamine
Phosphatidylinositol/PI	FA	FA	Inositol
Phosphatidylglycerol/PG	FA	FA	Glycerol
Phosphatidic acid/PA	FA	FA	H
Ceramide/Cer	LCB^d^	FA	H
Sphingomyelin/SM	LCB	FA	Phosphocholine
Glycosphingolipids/GSL^e^	LCB	FA	Carbohydrates
Cholesterol/CHOL	Structure shown in Fig. 1		

^a^*FA*, fatty acyl chain. ^b^Lysolipids may be present in all classes listed in this table (except for cholesterol), but are for simplicity shown for PC only. ^c^Ether-linked lipids (see Fig. 1) may be present in all glycerophospholipid classes shown (PC, PS, PE, PI, PG, and PA), but are for simplicity shown for PC only. Ether lipids with an alkyl chain is abbreviated as exemplified for PC-O and ether lipids with an alkenyl chain is abbreviated PC-P (the alkenyl ether phospholipids are often called plamalogens). ^d^*LCB*, long-chain base (see Fig. 1). ^e^The GSLs contain many different classes with a large variation in the carbohydrate structures (see [7] for an overview of these classes)

**Fig. 1:**
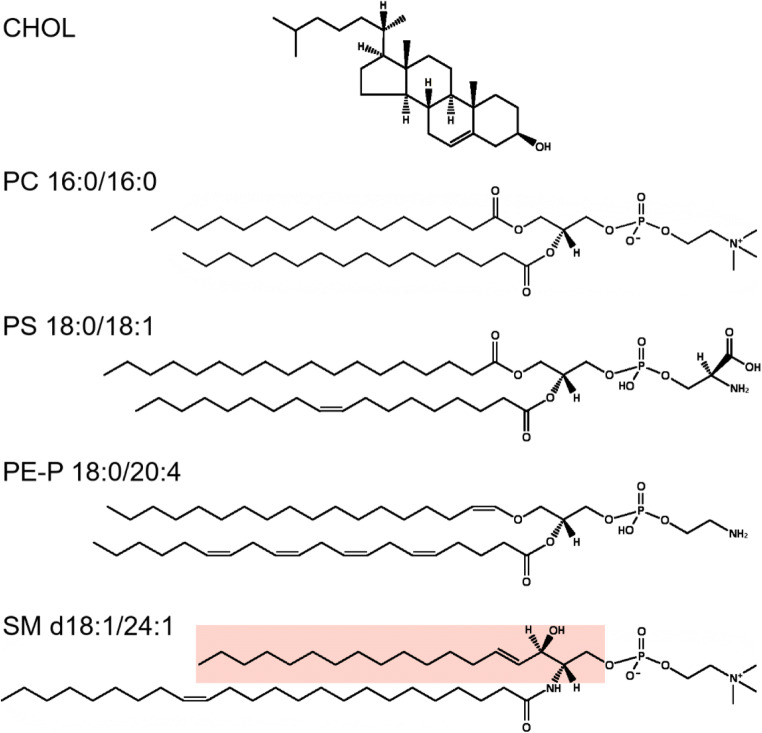
Illustrations of some lipid structures. Cholesterol is shown on the top followed by PC16:0/16:0, an example of a phospholipid with two saturated fatty acyl chains, which although much used in model membranes is not very common in biological samples. PS 18:0/18:1 is an example of a phospholipid with one saturated and one unsaturated fatty acyl chain, which is a very common combination, and this PS species is the most common PS species in many cells. Note that the unsaturated fatty acyl chains most often are found in the *sn-2* position and that all double bonds in phospholipids are in a *cis*-configuration. PE-P 18:0/20:4 is an example of an ether lipid with an alkenyl chain, i.e., a plasmalogen. The ether lipids often contain polyunsaturated fatty acyl groups in the *sn-2* position. Since all double bonds are in the *cis*-configuration, the polyunsaturated fatty acyl groups will “curl back” towards their head groups and not penetrate into the opposite leaflet even when containing as many carbon atoms as C20:4 (arachidonic acid) or C22:6 (DHA). The sphingolipid SM d18:1/24:1 is shown with the sphingosine part marked in pink. Note that the very long-chain N-amidated fatty acyl chain with 24 carbon atoms is so long that it can reach approximately halfway into the opposite leaflet. Glycosphingolipids contain very little of unsaturated fatty acyl chains, except for the 24:1 that is very common. The structures have been made by using the structure drawing tools available at Lipid Maps (https://lipidmaps.org/)

Most glycerophospholipids contain ester-linked fatty acyl groups, although ether-linked phospholipids are also common in most tissues (Fig. 1). The ether-linkage can be to an alkyl or alkenyl chain; the phospholipids with an alkenyl ether in the *sn-1* position are often called plasmalogens. The fatty acyl chains of the glycerophospholipids most often contain 16 or 18 carbon atoms with no or only a few double bonds in the *cis*-configuration. The unsaturated fatty acyl chains are most often C16:1, C18:1, or C18:2, although other fatty acyl chains like C20:4 (arachidonic acid) and C22:6 (docosahexanoic acid, DHA) are also common in some lipid classes; the unsaturated fatty acyl chains are most often present in the *sn-2* position [8].

Sphingolipids (Fig. 1) are synthesized from serine; different species within each class are obtained by having various N-amidated fatty acyl chains, i.e., fatty acyl chains bound to the N atom originating from serine. Glycosphingolipids frequently show a “bimodal” distribution of acyl chains, meaning that they contain mainly N-amidated C16:0 as the shortest species and C22–24 as the longest species. Glycosphingolipids normally contain very little of N-amidated fatty acyl chains with 18 or 20 carbon atoms, and they also contain very little of unsaturated fatty acyl chains, except for C24:1 species which may contribute even more than 50% of the total species of the class [9]. The reason for this bimodal distribution of glycosphingolipid species is not known, but it should be noted that the species containing N-amidated C16:0 will reach approximately to the middle of the bilayers (similar to that obtained by most glycerophospholipids), whereas the sphingolipids containing 24 carbon atoms could reach approximately halfway into the other leaflet, thus giving a flexibility for different strength of interactions between the sphingolipids in one leaflet and the lipids in the other leaflet [10].

Lipid rafts have for many years been described to be enriched in CHOL and sphingolipids and to be important for cellular signaling and the structure of cellular membranes [3, 4, 6]. In the present article, we describe the status for what is known about lipids in lipid rafts, the role of lipid species in leaflet interactions (caused by interdigitation) and membrane formation, and model systems used to study these structures. As discussed below, we find it useful and interesting to study exosomes as a model for cellular membranes. The exosomes are small vesicles of 50–150 nm which are released from cells by fusion of multivesicular bodies (late endosomes with intraluminal vesicles) with the PM. Importantly, exosomes contain just one lipid bilayer, and the lipid composition of exosomes is very similar to that reported for lipid rafts [11]. In this article, we discuss cellular uptake and transcytosis *via* caveolae, but we do not focus on the recently described role of caveolae regulating membrane mechanical stress [12]. We also briefly discuss caveolin, cavin, and flotillin, as scaffolding proteins in membrane organization.

## Studies of membrane lipids and leaflet interdigitation

### Distribution of CHOL in the PM and lipid rafts

CHOL constitutes 30–40 mol% of the lipids in the PM, and there may be an even higher fraction of CHOL in lipid rafts and exosomes [11, 13, 14]. Many groups have studied interactions between CHOL and other membrane lipids, including how CHOL is distributed between the two leaflets. The outcome of such studies varies a lot depending upon which method was used; some studies indicate that CHOL is similarly distributed between the leaflets, whereas other studies indicate that CHOL is mainly present either in the outer or inner leaflet of the PM. The different conclusions drawn from such studies strongly suggest that some of the methods used cannot be trusted, and we refer to an excellent review article where this controversial issue is summarized [15]. Based on the very high content of CHOL in some membranes, we find it difficult to understand how CHOL can be located mainly in one of the two leaflets, as we recently have discussed [10].

Further information about the importance of CHOL in cellular membranes is covered in several sections below. This includes the importance of CHOL for formation of lipid rafts, for membrane structure and interactions between the two membrane leaflets, and for invagination and endocytosis at the plasma membrane, and the role of CHOL in cancer and its possible use as a target for cancer treatment. For further information about the functions of CHOL in cell biology, we refer to a recent review article [16].

### PS in lipid rafts and caveolae

Caveolae have for many years been described to be enriched in sphingolipids and CHOL. In 2011, the groups of Grinstein and Parton observed, by using light microscopy and a PS-binding fluorescent probe together with electron microscopy and gold immune labeling of this probe, that PS clusters with a diameter of ~ 11 nm were present at the inner leaflet of the PM, and PS clusters were also observed at vesicular profiles (60–80 nm) with the morphology of caveolae [17]. Recently, it was shown that PS in fact dictates the assembly and dynamics of caveolae [18]. The enrichment of sphingolipids, CHOL, and clusters of PS in caveolae suggests that interactions between the two membrane leaflets are important for the formation of caveolae.

**Fig. 2:**
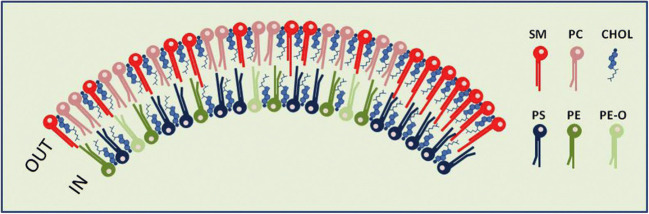
Schematic model of the lipid bilayer of exosomes, which have a lipid composition similar to lipid rafts, e.g., with a high content of SM and CHOL. The number of lipid molecules (excluding CHOL) shown in the outer (29) and inner (21) leaflet is close to the ratio of 1.36 for the outer and inner surface of exosomes with an outer diameter of 70 nm. The lipid composition of the membrane in this simplified illustration is based on the quantitative lipidomic data reported for 22 lipid classes of exosomes excreted from PC-3 cells [20], i.e.,16 SM, 13 PC, 12 PS, 6 PE, 3 PE-O (PE ethers), and 39 molecules of cholesterol (assuming a close to symmetric distribution of cholesterol between the two leaflets). In the right part of the membrane, a possible handshaking between the very-long-chain sphingolipids in the outer leaflet and PS 18:0/18:1 in the inner leaflet in the presence of cholesterol is illustrated. In the rest of the membrane, the lipids are distributed more or less evenly. Nine out of the 16 SM molecules shown contain a very-long-chain N-amidated fatty acyl chain in accordance with the data published. The figure is reproduced from [11]

We have earlier discussed [11] that membrane leaflet interactions between the two leaflets can take place in exosomes, which also contain high amounts of CHOL, sphingolipids, and PS, and show several similarities in their lipid composition with lipid rafts. Interdigitation caused by interactions between PS 18:0/18:1 and the very long–chain sphingolipid SM d18:1/24:0 in the presence of CHOL was shown by molecular dynamic simulation studies [19], which were performed following the observation that the PS 18:0/18:1, the very long–chain sphingolipids, and CHOL were all enriched to a similar extent in exosomes released from PC-3 cells [20]. These observations made us make a schematic model of the lipid bilayer of exosomes [11], here shown as Fig. 2. Exosomes are useful to study regarding their composition of lipids and interactions between the two membrane leaflets as these vesicles contain just one membrane, not the mixture of membranes found in cells. In addition, it is likely that there are large similarities between the lipid raft structures found in exosomes and in the PM [11]. Interesting in this connection is the remarkably similar enrichment of CHOL, SM, and PS from CHO cells to detergent-resistant membranes (DRMs) as well as rafts isolated without detergent [21] as the enrichments observed for these lipid classes in exosomes excreted from PC-3 cells [20]. In a recent review, we discussed the role of PS18:0/18:1 in membrane function and showed that data published from several laboratories indicate that this lipid species has an important function in coupling the two leaflets due to interactions with sphingolipids and CHOL [10].

Fairn and coworkers have during the last years published several interesting studies regarding the importance of PS in cells and the interactions between PS and CHOL [22]. They showed that the transbilayer distribution of CHOL was disturbed in PSB-2 cells, which have only 11% of wild-type PS synthase activity resulting in 80% less of PS than the original CHO cells, in the way that more CHOL was present in the outer leaflet without any changes in the total CHOL in the PSB-2 cells [23]. Moreover, the balance between CHOL in the two leaflets was normalized following addition of PS to the medium, but not by adding PE or PC. They also demonstrated that phase separation of CHOL and PS was obtained in giant unilamellar liposomes when using PS 18:0/18:1 (i.e., the same PS species as discussed above), but not when using PS 16:0/18:1, PS 16:0/18:2, or PS 18:1/18:1. Furthermore, they showed that PS 18:0/18:1 gave a better shielding of CHOL from being oxidized by cholesterol oxidase than PC 18:0/18:1, PE 18:0/18:1, PA 18:0/18:1, egg SM, and brain PI(4,5)P2 [23], providing further evidence for the interactions between CHOL and PS, and especially with PS 18:0/18:1.

It should be noted that in the study of exosomes released from PC-3 cells, a relative enrichment of lipids containing 18:0/18:1 species was reported not only for PS but also for PE, PI, and DAG [20]. Very recently, Mücksch et al. [24] reported quantification of 478 lipid species from 25 different lipid classes of HIV-1 particles released from MT-4 cells and the PM isolated from these cells. They concluded that there was enrichment from the PM to HIV particles of typical raft-like lipids such as SM, CHOL, and PS, with PS 18:0/18:1 constituting approximately 40% of the total PS species in these preparations. They also quantified species of mono-, di-, and tri-phosphorylated PI (i.e., PIP, PIP2, and PIP3) in the samples (to our knowledge, this is the first quantification of these lipids in lipid raft or exosome preparations). Remarkably, they found an increase in the relative amount of all these phosphorylated PI classes in HIV particles, whereas there was much less of the non-phosphorylated PI in HIV particles than in the PM (there is also much less PI in the exosomes released from PC-3 cells than in the mother cells [20]). It should also be noted that they found 36:1 (most likely 18:0/18:1) to be the dominant species together with 38:4 (most likely 18:0/20:4) in all PI classes (PI, PIP, PIP2, and PIP3). They speculated that the 36:1 species of these classes contribute to transbilayer coupling in a way similar to that described above for PS 18:0/18:1, although the sum of the four PI classes together contains much less of 18:0/18:1 species than PS 18:0/18:1 in these membranes.

While most discussions about lipid rafts focus on sphingolipid and CHOL rich domains in the outer leaflet of the PM, it is still not known whether lipid rafts can be created in one leaflet only. It should be noted that Prior et al. reported that also the inner leaflet of the PM contained a complex mosaic of discrete microdomains of 44 nm that occupied 35% of the inner leaflet surface [25]. That conclusion was based on observation of a CHOL-dependent clustering of a palmitoylated and farnesylated CAAX motif, which was stated to be a marker of inner leaflet lipid rafts [25]. However, it is not known whether there is a corresponding change in the outer leaflet. It should be noted that the group of Mayor has described transbilayer interactions between GPI-anchored proteins in the outer leaflet of the PM and PS in the inner leaflet [26]. In summary, more studies are needed to characterize the domains formed in both leaflets, and the interleaflet coupling.

### K-Ras and binding to PS in the PM

There are several isoforms of Ras GTPases (H-Ras, N-Ras, and K-Ras) which are ubiquitously expressed in mammalian cells and play critical roles in transducing extracellular signals. Although these isoforms are highly homologous, they generate different signal outputs [25]. Mutations in these isoforms are common in human cancers, with K-Ras mutations being the most prevalent Ras isoforms found in cancer [27]. K-Ras can form nanoclusters at the PM and these clusters are essential for K-Ras signal transduction. The distribution of K-Ras seems to depend on different posttranslational modifications such as phosphorylation and farnesylation, and K-Ras clustering is supported by farnesylation. While we consider that it is not clear whether K-Ras is mainly outside lipid rafts or also localized to lipid rafts for shorter periods, we comment upon the binding of K-Ras to the membrane in the following section.

The group of Hancock has published several interesting articles about K-Ras binding to the PM (for a review, see [27]). They showed that K-Ras nanoclusters at the PM colocalized with markers of PS, but not with markers of PIP2 [28]. The preferential binding to PS was shown to be due to a hexa-lysine polybasic domain close to the C-terminal farnesyl methyl ether on Cys185, a modification essential for membrane binding. Various treatments resulting in reduced amounts of PS in the PM caused loss of K-Ras from the PM and reduced clustering at the PM of the remaining K-Ras. Supplementation of exogenous PS, but not other lipids such as PIP2, PE, or PC, restored the K-Ras binding and clustering at the PM. Interestingly, this nanoclustering was restored only by adding PS species with one saturated and one unsaturated fatty acyl group (PS 18:0/18:1 or PS 16:0/18:1), and not when adding PS species with two saturated or two unsaturated fatty acyl chains [28]. Thus, these data emphasize the importance of specific PS species in binding and nanoclustering of K-Ras.

### Phospholipids with two saturated fatty acyl chains

Lipid rafts have for many years been stated to be enriched in saturated phospholipids, and most models of lipid rafts contain phospholipids with two saturated fatty acyl chains. We recently challenged this view [10] since the phospholipid composition published for lipid rafts [21, 29], the apical raft-like membrane of MDCK cells [14] and different cell lines contain very little of lipids with two saturated fatty acyl chains [10]. In an early and very detailed study of the lipid composition of DRMs it was stated that “a phospholipid was deemed saturated if containing no more than one double bond” [21]. This statement may have been overlooked as lipid rafts are frequently described to contain phospholipids with two saturated fatty acyl chains. Also, the higher lipid order measured in rafts compared with other parts of the PM [6, 30] may have contributed to this view; however, an increased lipid order might just be due to an enrichment of sphingolipids and CHOL, or reduction of phospholipids with polyunsaturated fatty acyl chains. Further quantitative lipidomic studies of the PM from cell lines with a varying content of caveolae would be useful to answer if phospholipids with two saturated fatty acyl chains are important constituents of lipid rafts. If that was the case, one would expect a larger fraction of the disaturated lipids in the PM of cells where caveolae constitute 50% or more of membrane surface area.

Purification of lipid rafts is a challenge, and it has been shown that the protein and lipid content of DRMs, which are often used to mimic lipid rafts, depends on the method used for their isolation [31]. Nevertheless, the lipid content of DRMs may at least to some degree reflect the lipid composition of lipid rafts. Since as much as 70% of giant plasma membrane vesicles (GPMVs) derived from fibroblasts has been reported to be composed of lipid rafts (based on staining with rhodamine-PE, which is claimed to be a lipid order domain marker) [32], quantitative lipidomic studies of these vesicles may provide important information about this issue.

### Ether lipids

Although ether lipids have been estimated to represent 18% of total phospholipids in humans [33], they have so far not obtained much attention, and they are still hardly mentioned in text books. Ether lipids have been reported to be involved in membrane trafficking, cell signaling, to possibly function as cellular antioxidants and to be enriched in cancer cells; we refer to the following review articles regarding ether lipids [33–36]. Importantly, the ether lipids are required for generation of the alkyl-containing glycosylphosphatidylinositol (GPI)-anchored proteins, which can be present in lipid rafts [26, 37].

Regarding membrane structure and lipid rafts, it is interesting that the ether lipids behave differently from their corresponding ester-linked analogs as to how the hydrophobic chains enter into membranes. The alkyl/alkenyl part of the ether lipids seems to enter perpendicularly into the membrane, whereas in phospholipids with fatty acyl groups, the two first carbon atoms in the *sn-1* position are almost parallel to the plasma membrane, while the rest of the acyl chain bends into the membrane. This knowledge was first obtained by using magnetic resonance spectroscopy comparing PC with two acyl chains and PC alkenyl ethers [38]. Atomistic molecular dynamic simulation studies were used to demonstrate a similar behavior of PE alkenyl ethers, including that the presence of PE ethers resulted in a more densely packed and thicker bilayer than PE with two fatty acyl chains [39]. Large amounts of ether lipids have been shown to be present in exosomes, and all PE species detected in exosomes purified from male human urine were in fact PE ethers (see [40] for a review about ether lipids in exosomes). Exosomes have a lipid composition with large amounts of SM and CHOL similarly to lipid rafts [11], and ether lipids have in a few studies been reported to be enriched in DRMs [29]. We believe that the mass spectrometry–based lipid analyses in the coming years will contribute to increasing our knowledge about ether lipids in cell membranes and lipid rafts.

### Lipid interdigitation and simulation studies

Molecular simulations studies have during recent years developed to be an important tool in studying membrane bilayers and interactions between the two leaflets (most often referred to as interdigitation). To our knowledge, the first molecular simulation study where asymmetric membrane models were used to study interdigitation based on quantitative lipid data of a biological membrane was a study by Rog et al. [19] based on the lipid composition of exosomes released from PC-3 cells [20]. This study revealed major differences in interdigitation when using asymmetric or symmetric models. The large interdigitation observed between SM d18:1/24:0 in one leaflet and PS 18:0/18:1 in the other leaflet was the only case where the interdigitation increased by adding CHOL to the two leaflets. Although the studies were performed based on lipidomics data for exosomes, these SM and PS species are as discussed above expected to be important constituents of lipid rafts, including caveolae.

For readers interested in such simulation studies, we refer to reviews discussing CHOL and sphingolipids in raft-like membranes [41] and the role of charged lipids in membrane structures [42], and two reviews discussing various theories and methods used for simulation studies [43, 44]. These reviews all stress the importance of using asymmetric models for such studies. It should be noted that it is now possible to make asymmetric liposomes [45]. We have recently discussed the importance of using both asymmetric models and lipid species commonly present in cell membranes to mimic such membranes in an optimal way in future studies with liposomes or in simulation studies [10].

Regarding molecular dynamic simulation studies directly related to caveolae, it has been reported that caveolin-1 can induce CHOL clustering not only in the inner membrane leaflet but also in the opposing leaflet, even when using symmetric bilayers of PC 16:0/16:0 in the absence or presence of 30% CHOL [46]. A positive membrane curvature was observed upon caveolin-1 binding to the CHOL-containing bilayers. These studies were performed using caveolin-1 with or without palmitoyl tails, and the authors concluded that the effect of the “palmitoyl tail is less clear and appears to increase the membrane contacts”. It would have been interesting to see a similar study performed with asymmetric membrane models and lipid species like those commonly found in cell membranes.

There are several studies where surprising, and not always consistent, results have been reported when model membranes were used to study interactions with viruses or toxins, illustrating that interpretation of such studies can be a challenge. As discussed below (Section 3.2), the SV40 virus is binding to the ganglioside GM1 and was believed to be taken up *via* caveolae. Ewers et al. investigated the effect of adding GM1 and PE species modified with the carbohydrate structure of GM1 on the SV40 virus-induced membrane invagination and infection [47]. Surprisingly, they observed a similar degree of SV40 infection following binding to PE 16:0/16:0 carrying the GM1 carbohydrate structure coupled to the ethanolamine group as after binding to GM1 (a mixture of d18:1/18:0 and d20:1/18:0), i.e., no differences were observed between the sphingolipid and glycerophospholipid part in the membrane. No infection was observed when shorter acyl chains, such as GM1 d18:1/8:0 or the modified PE 12:0/12:0, were used. They concluded that their analyses indicate that SV40, other polyoma viruses, and some bacterial toxins (Shiga toxin and cholera toxin) use glycosphingolipids with long acyl chains and pentameric binding proteins to induce PM curvature, thus directly promoting their endocytic uptake into cells.

The challenge of using model membranes and adding different lipid species is also illustrated by studies using Shiga toxin binding to Gb3. Semisynthetic C22:1 Gb3, but not C22:0, behaved like porcine kidney Gb3 and induced tubules in a liposome model [48], whereas it was the C22:0 Gb3 that gave similar results as the Gb3 mixture from porcine kidney when using a planar lipids model [49], thus revealing a model-dependent difference that so far has not been explained. Thus, although model membranes are popular to use for binding studies and in molecular dynamic simulation studies, one should be careful when drawing conclusions from such studies.

### Invaginations of membranes

Most endocytic processes involve after invagination of the plasma membrane, and several studies show that different treatment of cells that induce changes in membrane lipids, may also result in invaginations in the PM. Already in 1998, it was shown that treatment of fibroblasts with SMase induced ATP-independent endocytosis [50]. Later, it was reported that perturbation of the CHOL and sphingolipid balance in the PM, *via* extraction of CHOL using mβCD, or by using SMase to remove the phosphocholine headgroup of SM, resulted in formation of clusters of narrow tubular invaginations in several cell lines [51].

More than 20 years ago, Farge [52] reported increased endocytosis following addition of PS to K562 cells, and recently similar effects were reported in HeLa cells [53]. To monitor the fate of the added PS in HeLa cells, the authors used cells expressing a GFP-Lact C2 probe, a PS-binding molecule, and they found that the added PS was rapidly flipped to the cytosolic membrane leaflet, where it will increase the anionic charge and is hypothesized to affect membrane bending. In this study, the authors conclude that invaginations are formed not only by adding PS but also when CHOL is removed, thereby perturbing the balance between CHOL and PS [53]. Moreover, they found that endophilin and synaptojanin were recruited to these invaginations. They also reported that interactions between PS, the main phospholipid in the inner leaflet of the PM of most cells, and CHOL were important to control membrane curvature. They proposed that CHOL associates with PS to form nanodomains where the negatively charged headgroups of PS are kept sufficiently separated to limit curvature formation. They concluded that although the treatment used is clearly not physiological, the removal of CHOL illustrates the potential effects of increased charge density on membrane curvature. It should also be noted that there is a remarkable parallel amount of CHOL and PS in different cellular membranes, but further studies are needed to understand why [10, 53]. In summary, there are several ways to obtain invaginations in the PM or liposomes even without proteins described to be key components of, e.g., caveolae or clathrin-coated pits.

## Rafts with scaffolding proteins

### Caveolae

Caveolae (meaning “little caves”) are a type of lipid raft with a flask-formed shape. In some cells, like adipocytes, endothelial cells, and muscle cells, these structures are present at the PM at a very high density and may cover up to 50% of the PM area, whereas other cells contain only a few or no caveolae [12, 54]. Endothelial cells seem to have caveolae on both the apical and the basolateral sides [55, 56], whereas in polarized epithelial cells the caveolae are found on the basolateral side only [57, 58]. In addition to the high content of sphingolipids and CHOL, caveolae contain the proteins caveolin and cavin and each caveola has been reported to contain approx. 150 caveolin and 50 cavin molecules [54, 59, 60]. There are three forms of caveolin, which contain hydrophobic stretches that can penetrate into the lipid bilayer. No such hydrophobic stretches are found in the four forms of cavin, which bind to PS in the membrane. Remarkably, cavins can form a flexible and net-like protein mesh able to form polyhedral lattices when added to PS containing liposomes [59]. In 2012, two groups published that the protein EHD2 (an ATPase) had a stabilizing function at caveolae, preventing pinching off of these structures from the plasma membrane [61, 62]. More recently, it was reported that EHD2 is rapidly released from caveolae under mechanical stress, SUMOylated, and translocated to the nucleus, where it regulates the transcription of several genes including those coding for caveolae constituents [63].

A number of functions have been ascribed to caveolae. A quite recent one is their ability to protect cells against mechanical stress by stretching of the PM, which provides an explanation of the very high density of caveolae in some cell types, such as skeletal muscle, endothelial cells and adipocytes [12, 63–68]. Other functions include fatty acid [69] and Ca^2+^ [70] transport, as well as endocytosis as discussed below. Remarkably, the combination of lipids and proteins found in caveolae seems to be present not only in the curved caveolae structures but also in flat membrane structures, also believed to be lipid rafts [12, 71]. There is evidence that caveolin may function as a scaffolding protein to stabilize rafts in such domains at the PM as it has been reported to reduce uptake of membrane associated molecules (for reviews, see [72, 73]). There is still much to learn about interactions between these proteins and membrane lipids, but since cavin can bind to PS [61], caveolin can interact with CHOL and sphingolipids [46, 71, 74], PS can interact with CHOL [10, 53], and cavin interacts with caveolin [75, 76], it is clear that these proteins and lipids can form a network of importance for formation of caveolae.

### Endocytic uptake and transcytosis *via* caveolae

Whether caveolae are active in endocytosis or are merely stable structures contributing very little to endocytic uptake has been heavily debated for many years [77]. It has been reported that they may be important for uptake of many different types of molecules/substances, including protein toxins and viruses [78, 79], which were visualized by electron microscopy to localize to these structures. However, it is still not clear to which extent all these ligands are able to induce their own uptake from these structures, something which seems to be the case for the virus SV40 [79]. Visualization of a ligand could also be due to that it is trapped in caveolae. Although cholera toxin can accumulate in caveolae, it is also taken up by other endocytic mechanisms [80, 81]. It is important to keep in mind that the presence of a ligand in a certain structure such as caveolae does not imply that the ligand is internalized at a significant rate from the structure; it could be stuck there making it easier to visualize, e.g., by EM.

**Fig. 3:**
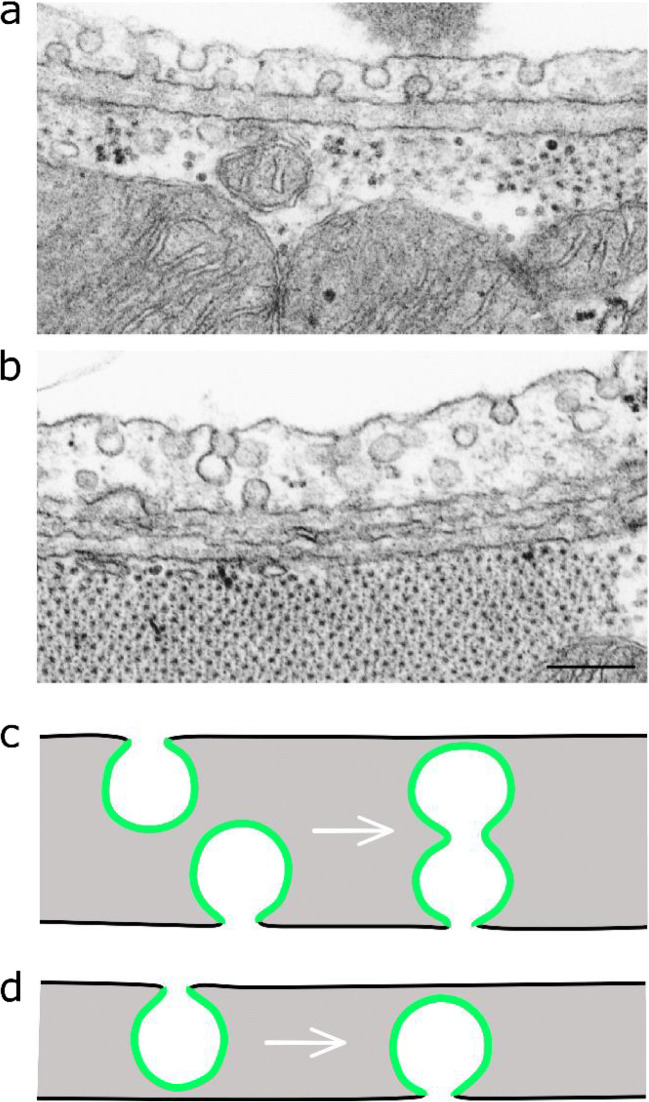
Two EM pictures (**a** and **b**) are showing examples of caveolae in cardiac capillary endothelium. These images are reproduced from van Deurs et al. [55] with permission from Elsevier. The scale bar in **b** is 200 nm. The two drawings at the bottom (**c** and **d**) illustrate the possibility for transendothelial transport *via* caveolae without formation of free intracellular vesicles

Caveolae seem to play an important role in uptake and transcytosis of albumin in various endothelial cells, where albumin binds to the albumin receptor gp60 (also called albondin) in caveolae [82, 83]. This mechanism is also reported to be involved in the transport of the active drug of Abraxane® (i.e., paclitaxel bound to albumin nanoparticles) [84], a product approved for treatment of several types of cancer (https://www.drugs.com/history/abraxane.html). It has also been suggested to exploit caveolae for delivery of other drugs [85–87], but to our knowledge, Abraxane® is the only such product that has reached the market. Although albumin is endocytosed *via* the gp60 receptor, it should be noted that mechanisms other than caveolae-mediated endocytosis may also be involved in its internalization as knock-out of caveolin-1 in pulmonary endothelial cells resulted in only 35% reduction of albumin uptake [88]. However, as discussed in the last paragraph of this section, there is a crosstalk between endocytic mechanisms so that a decrease in uptake from caveolae can increase other cellular uptake mechanisms. Furthermore, knock-out of caveolin in mice may increase the paracellular leakage of albumin and thereby complicate studies of the role of caveolin *in vivo* [89, 90]. The exact mechanism for how caveolae function in albumin transcytosis has not yet been clarified. This transcytosis process may not necessarily take place by caveolae endocytosis as a first step, and subsequent exocytosis at the basolateral side, but could happen without formation of free vesicles in very thin cells as illustrated in Fig. 3.

Caveolae-mediated endocytosis was originally described as the way SV40 viruses entered into cells; the virus was reported to enter vesicles called caveosomes where the content was not degraded in lysosomes but transported to the ER [79]. Later the same group reported that this virus was taken up even more efficiently by clathrin-independent and caveolin-1-independent endocytosis [91]. Almost 10 years ago, they concluded that caveosomes were an artefact due to over-expression of different constructs of caveolin-1, and wrote that the term caveosome should no longer be used [92]. Caveolae that do pinch off can at least in some cells fuse with normal early endocytic vesicles and thus be able to transfer material to lysosomes [92, 93]. The term caveosome has nevertheless survived in the nanomedicine field where one can still see investigators describing that they try to design nanoparticles, so they will enter caveolae, with the aim of avoiding lysosomal degradation. This demonstrates the need for collaboration between scientists in different areas.

Regarding studies of endocytic mechanisms, the lack of specific inhibitors for endocytic uptake is a challenge [94]. Wrong conclusions about these mechanisms and intracellular transport are frequently seen in fields such as nanobiotectechnology, even in high-impact journals. If the cellular uptake of a nanoparticle is inhibited by substances earlier reported to reduce endocytic uptake *via* caveolae, e.g., by reducing the CHOL levels in cells by adding methyl-β-cyclodextrin (mβCD) or other substances known to affect the CHOL levels, one often sees authors conclude that these nanoparticles are taken in *via* caveolae. However, reducing the CHOL levels in cells by extraction of CHOL using mβCD will not only affect the structure and uptake *via* caveolae, but also result in perturbation of clathrin-mediated endocytosis accompanied by formation of flat or shallow clathrin pits [95, 96]. Also, macropinocytosis is strongly reduced by mβCD extraction of CHOL [97, 98], and in fact most endocytic pathways can be expected to be affected by the CHOL level [72, 99].

Also, genistein is often used as a “specific” inhibitor of caveolae and it was indeed reported to inhibit SV40 induced vesicle formation from caveolae [100]. This does not, however, imply that this general inhibitor of tyrosine kinases can be used as a selective inhibitor of caveolae uptake. Genistein will also inhibit uptake *via* clathrin-mediated endocytosis of receptors which need tyrosine phosphorylation for accumulation in clathrin-coated pits, such as EGF receptor [101]. It is also reported to inhibit actin recruitment needed for clathrin-mediated endocytosis [102] and it may inhibit growth factor-induced ruffling and macropinocytosis, for instance caused by EGF [103]. To summarize some of the pitfalls in this field, we some years ago published a toolbox with some of the commonly used inhibitors [94].

The crosstalk between endocytic mechanisms makes it especially challenging to study these mechanisms. Not only can one and the same protein be involved in more than one mechanism; for instance, the small GTP-binding protein cdc42 is involved in the CLIC/GEEC pathway, macropinocytosis, and in FEME (fast endophilin-mediated endocytosis), but there is also a complex crosstalk between the various mechanisms [72, 99]. In 2010, Pagano and collaborators reported that there is a coregulation of cdc42-dependent uptake and caveolar endocytosis [104]. When phosphocaveolin is reduced, cdc42-GTP goes up, and fluid phase uptake is increased. A few years later, not only caveolin but also cavins were published to be involved in such a coregulation [105]. Thus, there has been an incredible development when it comes to our knowledge about endocytic mechanisms [72, 99].

### Flotillins

Another group of scaffolding proteins associated with lipid rafts is the flotillins (for a recent review, see [106]). The flotillins are like caveolins modified by fatty acyl chains, flotillin-1 with one palmitoyl chain and flotillin-2 with one palmitoyl and one myristoyl chain. The flotillins have been implicated in endocytosis without being directly associated with the endocytic process. They may rather be involved in pre-endocytic clustering of some ligands [72], and it has been suggested to call such cases for flotillin-assisted endocytosis [107]. On the other hand, flotillins may, like flat caveolin-associated membrane domains, stabilize receptors at the surface and prevent their uptake. For instance, the tyrosine kinase ErbB2, which is overexpressed in several breast cancers, is internalized and degraded upon knockdown of flotillin-1 or flotillin-2 [108, 109]. In agreement with the idea that rafts may prevent uptake of ErbB2 from the cell surface is the finding that lowering the cellular CHOL content by addition of lovostatin, an inhibitor of CHOL synthesis, will mediate internalization of ErbB2 [110]. Because of facilitated internalization, also ErbB2-mediated signaling is reduced [108]. Interestingly, tissue microarray analysis of biopsies from almost 200 breast cancer patients showed that flotillin-2 is a potential predictor of prognosis in breast cancer patients [108]. In the review by Liu et al. [106], there is a long list of publications about dysregulation of flotillins in different cancer types. Interestingly, it has been reported that siRNA-mediated knockdown of flotillin-1 decreased the caveolin-1 level in SK-CO15 cells, a human intestinal epithelial cell line [111]. The explanation for such a result is not clear, but the authors suggested that the reduction in caveolin-1 could be an indirect effect mediated by changes in cytoskeletal proteins. As shown for caveolin, phosphorylation of flotillin also seems to regulate its function. In the case of flotillin, dephosphorylation upregulates endothelial cell migration and angiogenesis [112].

## Lipid rafts, caveolae, and cancer

### More lipid rafts in cancer?

Cancer cells have been reported to exhibit altered lipid metabolism [113], and the role of lipid metabolism and the relation between the levels of certain lipids, e.g., CHOL, and cancer have been discussed for many years (for recent reviews, see [114, 115]). As mentioned above, also increased levels of saturated fatty acyl chains in membrane lipids have been coupled to the presence of more lipid rafts. In a recent study of the effects of lysophosphatidylcholine acyltransferase 1 (LPCAT1) in glioblastoma cells, the increase of disaturated PC species, especially PC 32:0, was taken as evidence for the importance of such lipids and lipid rafts for oncogenic signaling [116]. A commentary article concluded that these data in fact were “tying lipid rafts to oncogenic signaling” [117]. However, we are of the opinion that more data are needed to draw such a conclusion, because the depletion of LPCAT1 gave major changes in the whole lipidome, with many lipid species being changed similarly or even more than PC 32:0.

If there were more lipid rafts in cancer cells, one might expect higher levels of CHOL and SM. Several studies have revealed enhanced CHOL synthesis in cancer cells compared with normal cells, and high serum CHOL levels are associated with increased risk for several cancers such as breast, prostate, and colorectal cancers [114]. However, clinical studies with statins (inhibitors of CHOL synthesis) gave mixed results [115], and other tumor types, e.g., bladder and lung cancers, are not associated with increased CHOL levels [114].

Cells that are resistant to the drug doxorubicin have been reported to have a higher degree of structural order in the PM [118] and to have higher levels of SM and CHOL [119] than the corresponding doxorubicin-sensitive cells. Urothelial cancer cells contained a CHOL level that correlated with the cancerous transformation, and it was proposed that the CHOL/SM-rich membrane domains in these cancer cells should constitute a selective therapeutic target for elimination of these cells [120]. Also, vinblastine-resistant human leukemic lymphoblasts were reported to have more CHOL (and ether phospholipids) than the corresponding vinblastine-sensitive cells [121].

To our knowledge, the first study where extensive quantitative lipidomic analyses of cancer tissue biopsies were compared with adjacent normal tissue was published very recently [122]. A total of 342 species from 20 lipid classes were quantified in tumor samples and adjacent normal mucosa from patients with colorectal cancer (20 patients; 10 of each sex). The authors concluded that, in contrast to previous reports, the analyses of these colorectal cancer samples showed a lipid composition very similar to that of the normal colonic mucosa of the same individuals. The abundance of CHOL, SM, PC, PI, and PS was very similar. Some few differences were observed between the samples, such as an increase in lyso-PI in tumors (approx. 0.22% of total lipids in tumor and 0.14% in normal tissue), which may be important for the ability of this lipid to signal through the G-protein coupled receptor 55 (GRP55), and thus promote proliferation, migration, and invasion of cancer cells [123]. They also observed a small decrease in some ether lipids in advanced stages of colorectal cancer and speculated that this reduction was due to that cancer tissue contains higher levels of reactive oxygen species [124] and that ether lipids have been consumed to scavenge these radicals [33]. This study contains well documented quantitative lipidomic analyses, and similar studies should be performed with other types of cancer tissues and adjacent healthy tissues. It should, however, be noted that although this study reported detailed analyses of 20 lipid classes, the analyses did not include glycosphingolipids more complex than hexosylceramide, and several of the sphingosines or ceramides (sphingosine, sphingosine-1-phosphate, ceramide-1 phosphate, and dihydroceramide) discussed in Section 4.2.

More studies with quantitative lipidomic analyses are needed before final conclusions can be drawn regarding the level of CHOL, SM, and phospholipids with disaturated fatty acyl chains in cancer. One should also keep in mind that although there may not be an increase in the amount of lipid rafts in cancer cells, they may be qualitatively different, for instance, they may contain more glycosphingolipid species giving rise to transmembrane signaling (see Section 4.3).

### Perturbation in sphingolipid metabolism

Sphingolipids, which include many different lipid classes, have been reported to have a variety of important effects on physiology and disease (for review, see [125]). Regarding the present discussion, it should be noted that increased cellular levels of ceramides (Cer), dihydroceramides (dhCer), and sphingosines (Sph) are often connected with induction of cell cycle arrest and/or cell death, whereas increased levels of Cer-1-phosphate, Sph-1-phosphate, and the two glycosphingolipids, glucosylceramide and lactosylceramide, are often associated with increased cell survival, proliferation, and migration/invasion, i.e., events that are associated with cancer progression [125].

We refer to three review articles regarding discussions about the complicated effects of these lipids on cancer. Machala et al. [126] focus their discussion on colorectal cancer, where a number of enzymes involved in sphingolipid metabolism have been found to be deregulated in human tumors, in animal models and in human colon cancer cells *in vitro*. They advocate that an increased ratio of Sph-1-phosphate/Cer is often linked to cancer cell survival and progression, and that more attention also should be paid to the importance of glycosphingolipids. Don et al. [127] discuss changes in sphingolipid (including glycosphingolipid) metabolism in various types of cancer. They claim that the “simple notion of the balance” between Sph-1-phosphate and Cer as dictating cell survival is not in accordance with several recent studies, e.g., that selective Sph kinase 1 inhibitors do not affect cancer cell proliferation or survival, and the fact that several studies demonstrate higher Cer levels in some metastatic cancers. Moro et al. [128] discuss different aspects of Cer related to cancer, including using Cer as an anti-cancer agent, regulating Cer metabolism to suppress cancer, and using Cer as a biomarker.

In conclusion, there is a great need for more quantitative lipidomic studies that also include the Sph and Cer classes discussed above.

### Glycosphingolipid-containing rafts and signaling

Aberrant glycosylation appears to be a common feature in carcinogenesis and contributes to changes in cell signaling, growth, adherence, and motility [129]. Most human cancers have changes in the glycosphingolipid composition and metabolism, including the neutral globosides [130]. A number of cancer cells have increased levels of the globoside Gb3 on their plasma membrane [131], and targeting of different types of tumors for imaging or therapy has therefore been studied by using Gb3-binding ligands such as Shiga toxin or derivatives of this molecule [132–135]. Interestingly, it has been shown that metastasis of colon cancer cells is dependent on Gb3 [136]. The mechanisms responsible for this are not known but could be related to signaling induced upon crosslinking of glycosphingolipids. Both addition of Shiga toxin to cells as well as addition of antibodies against Gb3 can induce a flux of Ca^2+^, cytosolic tyrosine kinase activation, changes in life-time of clathrin-coated pits and activation of the phospholipase A2, with subsequent downstream effects in cells [137–140]. Similarly, cholera toxin has been shown to induce signaling [141], but since this toxin also can interact with proteins it is a more complex picture. The signaling induced by glycosphingolipid crosslinking could be raft-dependent and mediated *via* membrane leaflet interdigitation, since it is inhibited by addition of the CHOL-binding compound filipin [137]. It has previously been reported that addition of glycosphingolipids to cells can induce pinching off of caveolae [142] and this may not only be due to structural changes in the membrane as an increase in Src signaling was also reported.

Furthermore, the amount of the negatively charged gangliosides has been found to be increased in various pathological conditions, including several types of cancer, such as neuroblastoma, glioblastoma, melanoma, breast, and lung cancers [143]. This is especially the case for gangliosides containing more than one sialic acid bound to lactosylceramide. We refer to a recent review article regarding the structure and function of the gangliosides, and their involvement in cancer cell signaling, including their role in invasion and metastasis [143]. As discussed in that review, the effect of gangliosides is very complicated, and they can in fact have both positive and negative effects on signaling of receptor tyrosine kinases in cancer cells. It should also be noted that single-fluorescent-molecule imaging in live-cell PM has revealed a clear but transient colocalization and codiffusion of a fluorescent ganglioside analog with the fluorescently labeled GPI-anchored protein CD59 in a cholesterol-dependent manner, indicating that the raft domains are highly dynamic structures [144].

The changes in glycosphingolipid patterns observed in cancer cells are in several cell types shown to be associated with an increased activity of glucosylceramide synthase (GCS) which contributes to removal of Cer and gives a reduced tendency of a cell to undergo apoptosis [127]. Also, treatment with cancer drugs such as doxorubicin can activate transcription of GCS and thereby induce further changes in glycosphingolipid composition [127]. A challenge in cancer treatment is that such changes, and especially in the globoseries of glycosphingolipids, such as Gb3, are associated with increased expression of the human multidrug resistance 1 gene (MDR1) which encodes a drug transporter called P-glycoprotein (P-gp) [127, 145]. Thus, GCS is found to be overexpressed in many multidrug-resistant cancer cells. Liu et al. [145] found that silencing of GCS represses MDR1 expression and sensitizes cancer cells to drugs that would otherwise be removed by the drug transporter. However, also silencing of Gb3 synthase had a similar effect, showing the importance of Gb3 for induction of drug resistance. The authors reported that increased MDR1 expression was induced by signaling from glycosphingolipid-containing rafts; there was an activation of cSrc (formation of p-cSrc), leading to increased β-catenin in the nucleus and as a consequence increased MDR1 expression. Also, in breast cancer stem cells, Gb3 was found to activate the cSrc/β-catenin signaling pathway, Gb3 was found to correlate with the number of stem cells in breast cancer cell lines, and silencing of GCS was reported to kill the stem cells [146]. Thus, interfering with synthesis of glycosphingolipids or lipid raft signaling might be important in cancer therapy.

### Lipid therapy

Cancer cells are dependent on making new lipids for formation of more cell membrane and for lipid modification and membrane localization of proteins, including many oncogenic signaling enzymes. This and the upregulation of fatty acid synthase (FAS) in many cancers [147] have made researchers investigate whether inhibition of FAS could be used for cancer therapy. Many preclinical studies have been performed, but few have resulted in data being promising enough for these inhibitors to enter clinical studies. We refer to the following reviews [147, 148] regarding results obtained in earlier studies and an update of studies with new FAS inhibitors.

In agreement with their role as signaling hubs in cancer, lipid rafts can be targeted for therapy [149], and the term “membrane lipid therapy” was introduced some years ago [150]. The modified fatty acid 2-hydroxyoleic acid was introduced as an agent that should activate SM synthase and thus change biophysical properties of the membrane, leading to altered cell signaling in cancer cells and eventually to cell death [150, 151]. When we tested this substance on HeLa cells we did not observe any major changes in the lipidome, and no differences were observed for SM species in control cells or cells incubated with oleic acid or 2-hydroxyoleic acid [152]. We did, however, after treatment with 2-hydroxyoleic acid observe a reduced membrane fluidity, a rapid release of intracellular calcium, activation of several signaling pathways and a large increase in retrograde transport of the plant toxin ricin to the Golgi apparatus and the endoplasmic reticulum. Very recently, 2-hydroxyoleic acid was reported to cause cancer selective toxicity by uncoupling oxidative phosphorylation [153].

During the last years new lipid-derived compounds that apparently target rafts and raft-mediated signaling have been developed. These antitumor lipids are non-mutagenic and can inhibit tumor cell growth, metastasis, and angiogenesis, and several preclinical and clinical studies with antitumor lipids are ongoing, as reviewed by Zaremberg et al. [149]. Most of these lipids have structures somewhat like lyso-PC; they include ether analogues and also molecules not containing the glycerol backbone such as hexadecylphosphocholine; the structures of such lipids are shown in [149].

## Conclusions and key questions for future studies

More studies are needed to improve our understanding of the relationship between lipid structure and function, including membrane raft localization, effect of leaflet interdigitation, and transmembrane signaling. Detailed knowledge obtained with new methods and instruments will increase our ability to more concisely understand changes in cancer cells and to synthesize molecules able to target these cells. Studies of membranes with very different contents of caveolae or other lipid raft domains should contribute to answer which lipid species are present in these structures. To understand changes in lipid metabolism in cancer, it is important that more quantitative lipidomic studies, including measurements of glycosphingolipids, are performed on cancer tissue and adjacent normal tissue. Some key questions to answer in future studies are: (1) Which lipid species are involved in forming various forms of lipid rafts, including the curved and flat caveolae? (2) How do lipids interact with each other within the same leaflet and with lipids in the opposing leaflet and which species are involved in such interactions? (3) How do lipids and raft proteins interact to form a mixture of stable and dynamic lipid raft structures? (4) Ether lipids have so far obtained very little attention. Why does our body contain so much of the ether lipids and which role do these lipids play in lipid rafts? Finally, more studies with model membranes should be performed using asymmetric bilayers containing lipid species commonly present in cells.
